# Single‐Entity Electrocatalysis of Individual “Picked‐and‐Dropped” Co_3_O_4_ Nanoparticles on the Tip of a Carbon Nanoelectrode

**DOI:** 10.1002/anie.202014384

**Published:** 2020-12-14

**Authors:** Thomas Quast, Harshitha Barike Aiyappa, Sascha Saddeler, Patrick Wilde, Yen‐Ting Chen, Stephan Schulz, Wolfgang Schuhmann

**Affiliations:** ^1^ Analytical Chemistry—Center for Electrochemical Sciences (CES) Faculty of Chemistry and Biochemistry Ruhr University Bochum Universitätsstrasse 150 44780 Bochum Germany; ^2^ Inorganic Chemistry Faculty of Chemistry and Center for Nanointegration Duisburg-Essen (Cenide) University of Duisburg-Essen Universitätsstasse 7 45141 Essen Germany; ^3^ Center for Solvation Science (ZEMOS) Ruhr University Bochum Universitätsstrasse 150 44801 Bochum Germany

**Keywords:** carbon nanoelectrodes, Co_3_O_4_, electrocatalysis, single-entity electrochemistry, single-particle immobilization

## Abstract

Nano‐electrochemical tools to assess individual catalyst entities are critical to comprehend single‐entity measurements. The intrinsic electrocatalytic activity of an individual well‐defined Co_3_O_4_ nanoparticle supported on a carbon‐based nanoelectrode is determined by employing an efficient SEM‐controlled robotic technique for picking and placing a single catalyst particle onto a modified carbon nanoelectrode surface. The stable nanoassembly is microscopically investigated and subsequently electrochemically characterized. The hexagonal‐shaped Co_3_O_4_ nanoparticles demonstrate size‐dependent electrochemical activity and exhibit very high catalytic activity with a current density of up to 11.5 A cm^−2^ at 1.92 V (vs. RHE), and a turnover frequency of 532±100 s^−1^ at 1.92 V (vs. RHE) towards catalyzing the oxygen evolution reaction.

## Graphical abstract

A SEM‐controlled micromanipulator technique is proposed to pick single Co_3_O_4_ nanoparticles and place them on a modified carbon nanoelectrode surface. The obtained nanoassembly allows to extract the intrinsic oxygen evolution activity of the nanoparticle at industrially relevant current density devoid of film and ensemble effects. Post‐electrochemistry TEM analyses offer insight into structural transformations during electrocatalysis.

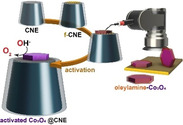

The real‐world electrochemical system is typically heterogeneous and comprised of a large ensemble of individual entities (atoms, molecules, ions, nanoparticles (NPs), cells) exhibiting varying shapes in a wide spectrum of sizes.^[1]^ Traditional electrochemical methods on a macroelectrode measure the averaged activities of the ensemble and the resulting electrochemical responses are often affected by a variety of interlinked system parameters, for example, film effect or local pH change at such ensembles. Hence, the electrochemical response is often too complex to deconvolute the catalytic performance, which prevents determination of the intrinsic activity of a catalyst material.^[2]^ In this regard, the development of tools and strategies capable of measuring and analyzing electrochemical reactions of individual entities remains central to the advancement of “single‐entity electrochemistry”.^[3]^ Nanoelectrochemical measurements exploit the nanometric dimensions of nanoelectrodes to study individual nano‐sized particles utilizing the high signal‐to‐noise ratio and high rate of mass transport at the nanoelectrodes to derive information about intrinsic properties of individual catalyst particles.^[4]^ In the case of freely diffusing catalyst nanoparticles, the electrochemical activity is measured by detecting the transient electrochemical current arising from the collision of nanoparticles with the nanoelectrode under potential control.[^5^, ^6^] The intrinsic activity parameters calculated using such nano‐impact amperometric analysis cannot provide information on the orientation of these particles during the collision event. Therefore, these experiments can only provide limited insight into the correlation of structure, orientation, and activity. Furthermore, the time‐resolved voltammetric analyses of such nano‐impact measurements necessitate a high potential scan rate owing to the short time of residence of the nanoparticle on the electrode.^[7]^ In contrast, mobile pipet‐based techniques such as scanning electrochemical cell microscopy (SECCM) rely on the combination of post‐experimental microscopic investigation of the probed surface with the locally obtained electrochemical response to establish structure–property relationships.^[8]^ In contrast, the ability to attach a pre‐selected well‐shaped single nanoparticle from a large ensemble and place it onto a nanoelectrode could facilitate high‐resolution imaging of such nanoassemblies in parallel with electrochemical analyses. Such measurements provide new opportunities in the field of structure–property correlation on the scale of single‐entity measurements.^[9]^ To date, numerous approaches have been adopted to attach specific nanoparticles onto a nanoelectrode surface, namely, electrodeposition,[^2^, ^10^] direct adsorption from the suspension onto the electrode surface,^[11]^ electrostatic attachment onto a charged pre‐deposited layer,^[12]^ and formation of covalent metal−C bonds via electrochemical reduction of the diazotized carbon surface.^[13]^ However, these approaches are laborious and offer a limited probability of obtaining exclusively a single nanoparticle on a nanoelectrode surface, a major pre‐requisite for ideal “single”‐entity measurements. Thus, the development of a versatile “go‐to” technique for immobilizing a single nanoparticle onto the nanoelectrode surface with greater ease, better control, and improved stability is a challenging scientific task to precisely evaluate structure–property correlations for single nanoparticles.^[7]^ Herein, a SEM‐controlled technique is employed to attach oxygen evolution reaction (OER)‐active spinel‐type Co_3_O_4_ nanoparticles onto modified carbon nanoelectrodes (CNEs) (Scheme 1). Specifically, hexagonal‐shaped crystalline Co_3_O_4_ nanoparticles of varying sizes are chosen as the catalyst system of interest. Moreover, methods to reinstate electrocatalytic activities of surfactant‐capped Co_3_O_4_ nanoparticles prior to their electrochemical activity evaluation are discussed. These methods include electron beam‐induced and heat‐induced surfactant removal. The extraction of intrinsic electrochemical activity parameters based on detailed microscopic investigations and structural assessment of individual catalytic units provides a deeper understanding of structure–property correlations.

**Scheme 1:**
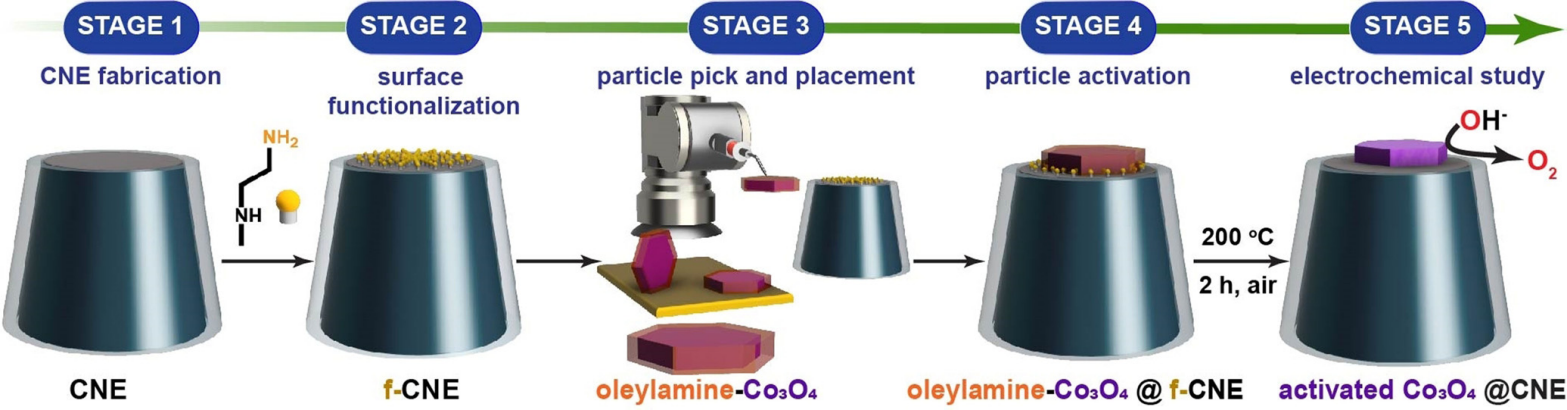
Flowchart illustrating the various stages involved in fabrication, activation, and evaluation of the Co_3_O_4_@CNE nanoassembly.

The hexagonal‐shaped nanoparticles of varying sizes (180–300 nm) are synthesized using a wet colloidal synthetic approach and oleylamine as the structure‐directing agent.^[14]^ The XRD pattern of the nanoparticles can be assigned to the planes of the Co_3_O_4_ spinel‐type structure (JCPDS card no. 01‐076‐1802) (Figure S1). No additional crystalline phases were observed as shown by Rietveld refinement, demonstrating the formation of a phase‐pure material. The CNEs are fabricated using laser‐pulled quartz capillaries and a double‐step pyrolysis protocol. A propane/butane gas mixture was used as the carbon source in an automated pyrolysis set‐up, as reported previously (details in Section 2, ESI).^[15]^ The obtained electrodes are further processed using a focused ion beam (FIB) to obtain CNEs with well‐defined electrode dimensions (Figure S2c). The scanning electron microscopy (SEM) investigation of the FIB‐processed CNEs indicates that the disk diameters range between 500 and 600 nm (Figure S2f–h). In order to improve the chemical compatibility of the oleylamine‐capped nanoparticles with the CNE, the electrodes are subsequently surface‐modified by a one‐step grafting of *N*‐Boc‐ethylenediamine (*tert*‐butyl *N*‐(2‐aminoethyl)carbamate) in anhydrous ethanol containing 0.1 m LiClO_4_ as supporting electrolyte.^[16]^ In a first step, the free amino group of *N*‐Boc‐ethylenediamine is oxidized by an anodic potential sweep from 0.2 to 1.8 V vs. Ag/AgCl/3 m KCl at a sweep rate of 50 mV s^−1^ ^[17]^ under subsequent coupling of the in situ generated primary amino cation radical to the CNE surface.^[18]^ The decrease in oxidation current during the second scan corresponds to the formation of a blocking layer that restricts the access of *N*‐Boc‐ethylenediamine species to the CNE surface (Figure S3a). The Boc protection groups are then removed by treatment with 4 m H_2_SO_4_. The surface blocking characteristics of the grafted layer are investigated by studying the redox conversion of [Ru(NH_3_)_6_]^3+^ before and after the surface modification (Figure S3c). Evidently, the surface blocking is too high using the potential sweep strategy and the time for the formation of the primary amino cation radical has to be significantly shortened.

We hence applied a single potential pulse (20 ms) to a potential of 1.5 V vs. Ag/AgCl/3 m KCl to obtain an optimal compromise between sufficient surface modification and negligible additional resistance. The redox conversion of [Ru(NH_3_)_6_]^3+^ before and after the surface modification (Figure S3d) does not show substantial deviation, demonstrating the accessibility of the modified CNE surface.

The hexagonal‐shaped Co_3_O_4_ particles are first drop‐coated onto an ultra‐flat gold wafer, from which they are then selected under SEM control. A micromanipulator arm, which is mounted on a motorized stage in the SEM chamber, is used to isolate the desired nanoparticle from the bulk sample (Figure S4). The selected nanoparticle is then picked up by gradually approaching the micromanipulator tip until contact with a speed ranging from 2 mm s^−1^ for coarse positioning to ≤5 nm per increment for fine positioning of the tip (Figure 1 a). Upon contact, the tip is gently pressed against the nanoparticle. After the nanoparticle is picked up, the tip is then moved under continuous SEM visualization so that the tip‐attached nanoparticle is located several mm above the CNE surface. In a slow approach, the nanoparticle is then carefully placed onto the modified CNE surface to ultimately transfer the nanoparticle to the CNE surface under formation of a Co_3_O_4_@f‐CNE nanoassembly (Figure 1 f+h). The physical picking‐and‐placing method provides a distinct advantage since it is possible to actively control the orientation of the nanoparticle on the CNE surface (Figure 1 c). The Co_3_O_4_@f‐CNE nanoassemblies further facilitate a detailed TEM investigation using a customized TEM CNE holder (Figure 1 g+i). The EDS elemental maps confirm the attachment of hexagonal‐shaped Co_3_O_4_ nanoparticles at the tips of CNEs. The particle placement strategy is verified multiple times to ensure the reproducibility of this approach.

**Figure 1:**
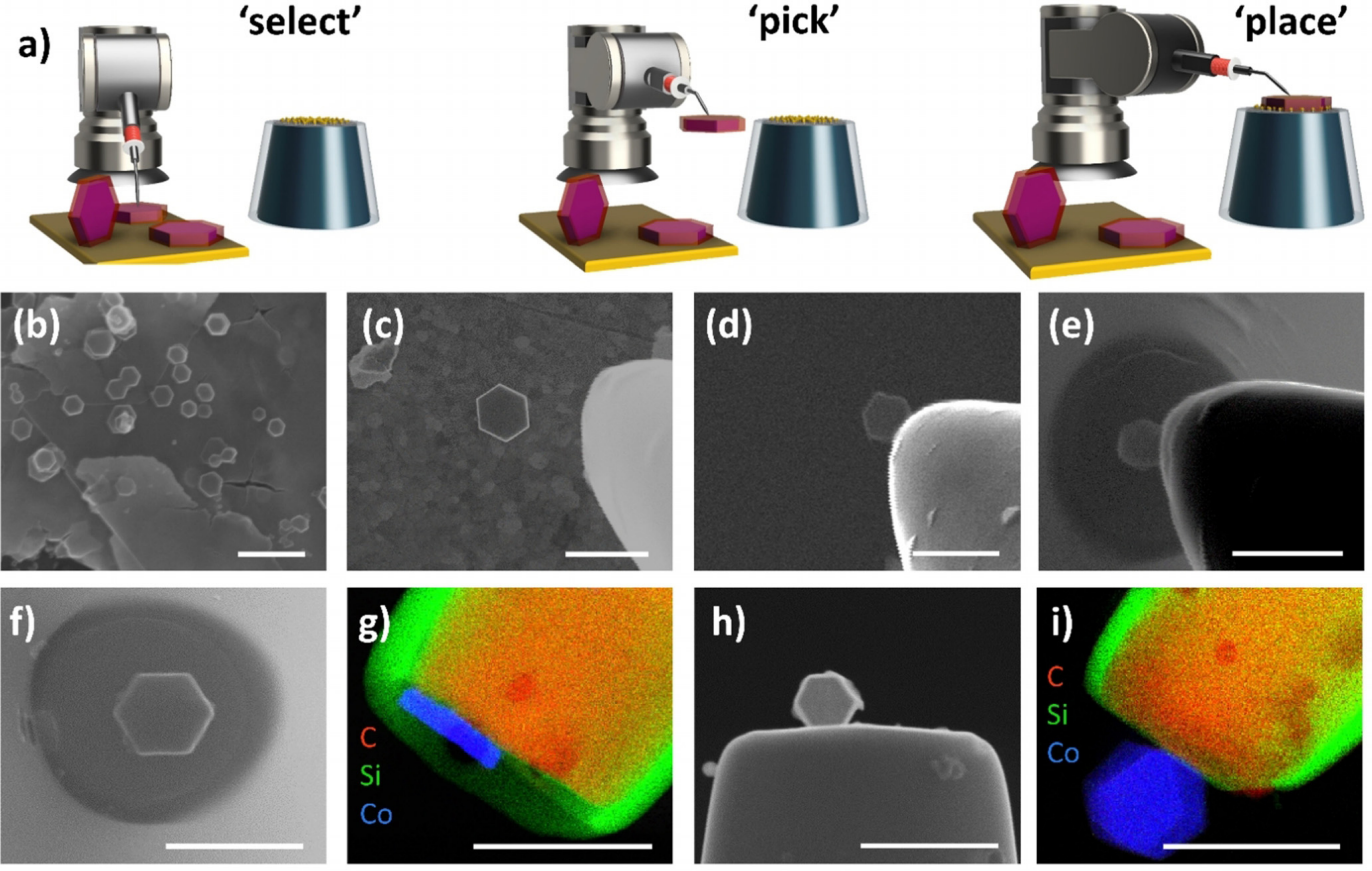
a) Illustration and b–e) SEM snapshots of the various stages involved in the picking and placing of individual hexagonal Co_3_O_4_ particles onto the modified CNE; f+h) SEM images of Co_3_O_4_@CNE nanoassemblies with different orientations of the particles on the modified CNE. g+i) Corresponding elemental maps (EDS‐TEM) of the nanoassemblies shown in (f+h). Scale bars equal 500 nm.

Electrochemical investigation of a freshly fabricated nanoassembly reveals that the oleylamine‐capped nanoparticle is catalytically inactive towards the OER (Figure S5). Interestingly, a second electrochemical investigation after TEM analysis indicates a significant increase of the OER activity of the same nanoassembly (Figure S5b), which is attributed to the at least partial removal of the oleylamine capping layer due to interaction with the high energy electron beam.^[19]^ Such a limitation is widely known in the case of surfactant‐capped catalyst nanoparticles.^[20]^ Various methods including thermal annealing, UV‐ozone irradiation, solvent washing are reported to remove oleylamine from nanoparticle surfaces.^[21]^ We hence employed a comparably mild activation protocol, which involves low‐temperature annealing to remove the oleylamine from the picked and placed particle surface (Figure S5d). TEM investigations of the nanoassemblies after annealing at 200 °C for 2 h in air confirmed the retention of the size and morphology of the nanoparticle.

The activated Co_3_O_4_@CNE nanoassembly is thereafter investigated for its OER activity in a two‐electrode configuration using an Ag/AgCl/3 m KCl as reference/counter electrode (for further details, see ESI). The electrocatalytic activity is initially evaluated by linear sweep voltammograms at a scan rate of 200 mV s^−1^ in 1 m KOH. The hexagonal‐shaped Co_3_O_4_ nanoparticles are found to exhibit size‐dependent OER activity (Figure 2 a). The measured absolute currents range from 7 to 13 nA. Advantageously, these nanoassemblies allow for precise determination of the geometric surface area by means of SEM. Considering the hexagonal shape of the Co_3_O_4_ nanoparticle and the mode of its attachment on the CNE surface, the intrinsic activity expressed as current density or turnover frequency (TOF) can be derived. Strikingly, analyses of three independent nanoassemblies (Figure 2 b) yield almost identical catalytic OER responses after normalization to the exposed particle surface area, which highlights the reproducibility of the proposed particle placing protocol (Figure 2 c). The OER activity evaluation demonstrates an extremely high OER activity of the catalyst in 1 m KOH, reaching current densities at 1.92 V vs. RHE of up to 11.5 A cm^−2^ (details in Section 6, ESI), a value which exceeds more than 20 times the applied current density in a commercial alkaline electrolyzer of 0.5 A cm^−2^.^[22]^

**Figure 2:**
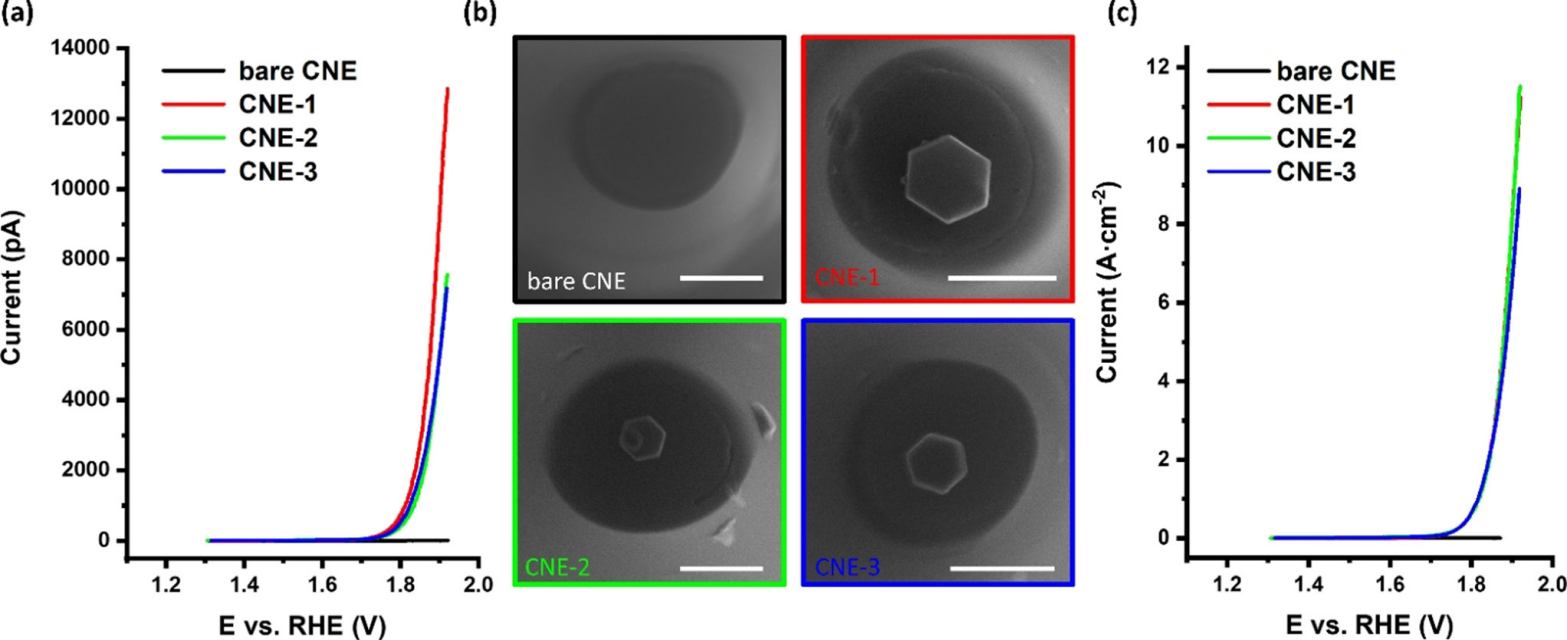
a) Linear sweep voltammograms (LSVs) of three independent Co_3_O_4_@CNE nanoassemblies, b) corresponding SEM images (color code of the frames is equal to the color code shown in (a) and (c)), and c) LSVs shown in (a) normalized by the electrochemical active surface area. Electrochemical measurements were recorded in 1 m KOH with a scan rate of 200 mV s^−1^. Bare CNE (black), CNE‐1 (red), CNE‐2 (green), and CNE‐3 (blue). Scale bars equal 400 nm.

The generally used value for comparing different catalyst materials in rotating disk electrode experiments of 10 mA cm^−2^ is reached at 1.66 V vs. RHE. Since ensemble effects, for example, the local pH change at the electrode, do not influence the reaction significantly at this low current density, this value is in very good agreement with the RDE data collected previously.^[14]^ Even though the TOF calculation was done under the very conservative assumption that all Co atoms of the Co_3_O_4_ NP are contributing, a considerable high TOF value of 532±100 s^−1^ at 1.92 V vs. RHE, compared to our previous work (29.7 s^−1^),^[9]^ was determined considering that the Co atoms in the bulk of the particle do not participate in the catalytic conversion. On the other hand, calculating the TOF considering only the surface Co atoms (Figure S7) as active sites leads to significantly higher TOF values, although this holds the risk of an overestimation. However, doing so a TOF value of up to 2.67×10^4^±5000 s^−1^ was derived. This value is about one order of magnitude lower than that reported previously using nanoimpact studies.^[6]^ Measurements at such high current densities are only possible at nanoelectrodes due to the increased mass transport allowing fast removal of the formed O_2_ as well as replenishing of the consumed OH^−^ ions.

Chronoamperometry was additionally performed in 0.1 m KOH at a constant potential of +1.77 V vs. RHE to assess the particle retention stability of the nanoassemblies during long‐term measurements. A stable current is obtained for those nanoparticles which were placed on amino‐group modified CNE surfaces while on non‐modified CNEs the particles were lost from the surface (Figure S9).

High‐resolution TEM images (Figure 3 a–d) and corresponding fast Fourier transform (FFT) images of pristine Co_3_O_4_ particles reveal the presence of lattice fringes with an inter‐planar spacing of 2.83 Å corresponding to the (220) planes of the crystal structure of Co_3_O_4_ spinel (Figure 3 e). However, post‐electrocatalytic TEM images of the Co_3_O_4_@CNE nanoassembly (Figure 3 b) indicate drastic structural transformations within the particle (Figure 3 d). High‐resolution TEM images reveal the emergence of amorphous domains within the particle, which is further confirmed by the diffuse FFT pattern (Figure 3 f). The residual crystal structure of the Co_3_O_4_ particle can be observed in the FFT shown in Figure 3 f where an inter‐planar spacing of 1.43 Å corresponding to the crystal plane (440) can be extracted. The EDS elemental maps of the particle indicate an increase in the oxygen content at the nanoparticle surface where the amorphous region emerged (Figure S8), which suggests the presence of oxygenated species. Although the chemical state of the formed amorphous region of the particle could not be confirmed, the loss of crystallinity in combination with the oxidation of the particle shell during electrocatalysis is expected to result from the generation of Co hydroxide/oxyhydroxide species, which are reportedly the catalytically active sites in Co‐based systems at the potentials invoking the OER.^[23]^

**Figure 3:**
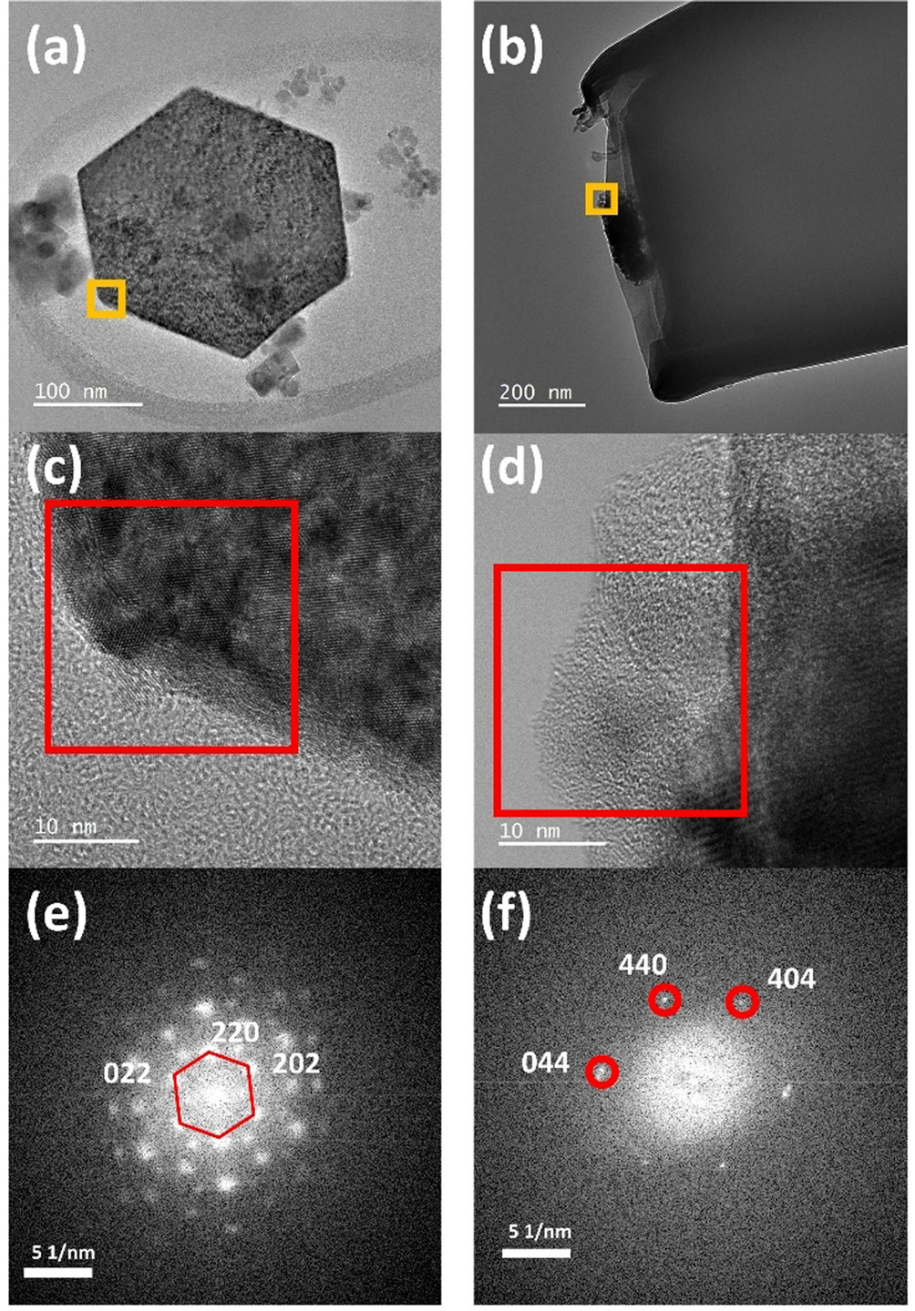
a+c) HRTEM images and e) corresponding FFT pattern of a pristine crystalline hexagonal Co_3_O_4_ particle; b+d) HRTEM images and f) corresponding FFT pattern of the particle post‐electrocatalytic test.

In summary, a versatile SEM‐controlled micromanipulator strategy is proposed and utilized to selectively pick and place an individual hexagonal‐shaped Co_3_O_4_ catalyst particle onto a chemically compatible CNE surface under formation of a robust Co_3_O_4_@CNE nanoassembly. Annealing at mild temperatures is used to remove the oleylamine capping layer and hence to activate the nanoparticle for the OER. The hemispherical diffusional flux at the nanoelectrode enables evaluation of the intrinsic OER activity of a single hexagonal Co_3_O_4_ particle at high mass transport rates under alkaline conditions. Strikingly, reproducible electrocatalytic responses—each amounting to a remarkably high TOF activity of 532±100 s^−1^ at 670 mV overpotential—are observed for three independently investigated particles. Furthermore, structural transformations during OER electrocatalysis are elucidated using HRTEM, confirming amorphization of the initially crystalline Co_3_O_4_ catalyst particle at the high anodic OER potentials. By using the proposed methodology we envisage that single‐entity analysis of catalyst materials has finally come to the point of reliable and comparatively facile sample preparation and characterization, which are critical for establishing structure–function relationships, a feature primarily desired for the rational design of catalysts for emerging energy technologies.

## Conflict of interest

The authors declare no conflict of interest.

## Supporting information

As a service to our authors and readers, this journal provides supporting information supplied by the authors. Such materials are peer reviewed and may be re‐organized for online delivery, but are not copy‐edited or typeset. Technical support issues arising from supporting information (other than missing files) should be addressed to the authors.

Supplementary
