# Supplementary material for: Single‐Entity Electrocatalysis of Individual “Picked‐and‐Dropped” Co3O4 Nanoparticles on the Tip of a Carbon Nanoelectrode
Source: Angew Chem Int Ed Engl. 2020 Dec 14;60(7):3576–80. doi: 10.1002/anie.202014384 (PMC7898714; doi:10.1002/anie.202014384)
Supplement: Supplementary file 1 — Supplementary [file ANIE-60-3576-s001.pdf]

Angewandte Chemie
Eine Zeitschrift der Gesellschaft Deutscher Chemiker

Supporting Information

Single-Entity Electrocatalysis of Individual “Picked-and-Dropped” Co_3O_4 Nanoparticles on the Tip of a Carbon Nanoelectrode

*Thomas Quast, Harshitha Barike Aiyappa, Sascha Saddeler, Patrick Wilde, Yen-Ting Chen, Stephan Schulz, and Wolfgang Schuhmann**

anie_202014384_sm_miscellaneous_information.pdf

Table of Contents

Section No.	Title	Page No.
	Experimental Procedures	3
1	Material Characterisation	4
2	Fabrication of carbon-based nanoelectrodes	5
3	Surface modification of the CNE	6
4	Micromanipulator set-up	7
5	Particle activation	8
6	Determination of the size-dependent electrochemical activity	9
7	TEM characterization post electrochemical study	12
8	Evaluation of the stability of the prepared nanoassembly	13

Experimental Procedures

Chemicals and materials

KOH (Fisher Chemical), N-Boc-ethylenediamine (>98%, Sigma-Aldrich), $LiClO_4$ (Sigma-Aldrich), Ethanol (VWR Chemicals), $[Ru(NH_3)_6]Cl_3$ (Sigma-Aldrich), KCl (Honeywell), oleylamine (OLA; ≥98%; Sigma-Aldrich), $Co(II)(NO_3)_2$ * 6 H_2O (99%; ABCR). The quartz capillaries with an outer diameter of 1.2 mm / inner diameter of 0.9 mm with a total length of 7.5 cm were purchased from Science Products.

Instruments

The quartz capillaries were pulled using a P-2000 laser puller (Sutter Instrument) using the following pulling parameters: HEAT=780, FILAMENT=4, VELOCITY=45, DELAY=130, and PULL=100. Scanning electron micrographs were recorded using a Quanta 3D ESEM (FEI) at 30 kV in the high vacuum mode. Electrostatic charging of the carbon nanoelectrodes was prevented by inserting a copper wire from the back end of the capillary, which is essential for an effective FIB milling process. Focused ion beam (FIB) milling of the as-prepared CNEs was performed using Quanta 3D ESEM, which is equipped with a Ga-FIB source. High-resolution transmission electron microscopy (HR-TEM) was performed using a JEOL microscope (JEM-2800) equipped with a Schottky-type emission source working at 200 kV, Gatan OneView camera (4kx4k, 25FPS) to obtain images with a resolution of 0.09 nm. Energy dispersive spectroscopy (EDS) elemental mapping was performed using double silicon drift detectors (SDD), with a solid angle of 0.98 steradians with a detection area of 100 mm^2. Electrochemical measurements were performed inside a Faraday cage in precleaned 1.0 M or 0.1 M KOH, which was placed inside another Faraday cage, using a ModuLab potentiostat from Solartron Analytical in a two-electrode configuration using a homemade double junction Ag/AgCl/3M KCl as both reference and counter electrode.

Results and Discussion

Section 1: Material characterisation

Co_3O_4 hexagonal platelets were synthesized following a previously published procedure.[1] Briefly, a solution of 2 mL of oleylamine (OLA) and 10 mL of EtOH was added dropwise to a stirred solution of 291 mg (1.00 mmol) of $Co(NO_3)_2{\cdot}6\ H_2O$ in 20 mL of EtOH. The resulting mixture was then transferred into a 50 mL stainless steel autoclave with a Teflon inset and heated in an electric furnace to 180° C for 15 h. The resulting precipitate was isolated via centrifugation (3000 rpm, 10 min), washed twice with EtOH and dried at ambient temperature. The powder was calcined at 300 °C in air for 1 h in order to obtain the spinel phase and to remove most of the organic surfactants. The removal of the surfactants by heat treatment can be observed in the Fourier-transform infrared spectra, where a complete elimination of the alkyl C-H stretch absorptions between 2850-2950 cm-1 after the calcination process was observed (Figure S1c). For the single entity electrochemical experiments the particles were re-suspended in ethanol using ultrasonication.

The X-ray diffractogram (XRD) shows the typical reflexes for the Co3O4 spinel-type structure, with the highest intensity for the (311) plane at 36.84°. No additional crystalline phases were observed as was supported by Rietveld refinement, demonstrating the formation of phase-pure material. A lattice parameter of a = 8.090 Å was determined for the hexagonal platelets which is in a good agreement with the reported values for Co3O4 (8.072 Å, PDF 01-076-1802). Additionally, the difference shows a slightly dominant surface termination of (111) planes, which is consistent with reports in the literature [2] and is formed by a combination of (220)- and (222)-planes.

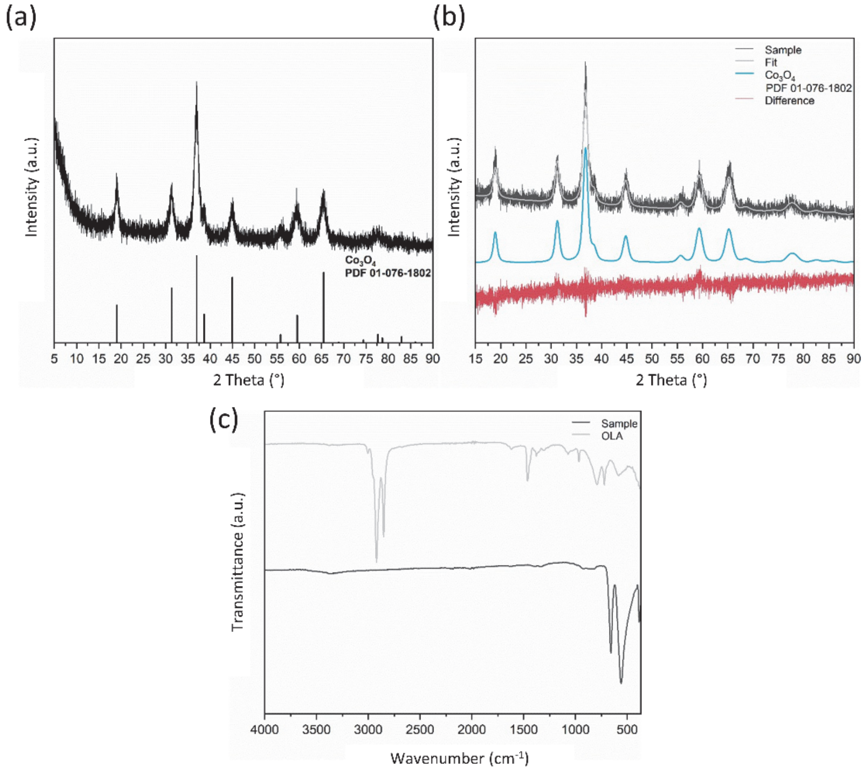

Figure S1: (a) Powder X-ray diffraction (PXRD) pattern of the hexagon-shaped Co_3O_4 NPs. **(b)** PXRD pattern of the synthesized NP (black) the calculated pattern (blue) and the obtained difference after subtraction of the calculated pattern from the as-synthesized. **(c)** FTIR measurement of the as-prepared NPs (grey) and the obtained spectra after calcination at 300 °C (black).

Section 2: Fabrication of carbon-based nanoelectrodes

Quartz glass capillaries were pulled using a CO_2-laser puller P2000, Sutter Instruments) to produce nanometre-sized quartz capillaries. In the subsequent step, the carbon-based nanoelectrodes were fabricated by deposition of carbon inside the quartz capillaries through controlled pyrolysis of a gas mixture composed of propane (Air Liquide, technical grade) and n-butane(Air Liquide, 99.5 %) under a protective argon counter-flow (Air Liquide, 99.999%, 50 mL/min) using a fully automatized set-up.[3] A two-step pyrolysis procedure was employed, where an initial temperature of 980 °C ±5 °C was used to deposit a thin, uniform and conductive carbon film along the inner surface of the quartz capillary. The second heating step was carried out using a peak temperature of 800 °C ±5 °C followed by a cool down phase over 35 s down to 475 °C to completely seal the carbon orifice of the capillary (Figure S3a).

The as-produced electrodes do not possess a perfect disk geometry (Figure S2b+e) which is essential for the particle placement. Therefore, these electrodes were further processed using focused ion beam (FIB) milling inside the scanning electron microscope. The FIB milling process was performed focusing a Ga^+-ion beam (Figure S2c) (100 pA current 30 kV acceleration voltage) for several seconds on the carbon nanoelectrode which yields a flat and neat carbon disk-shaped electrode (Figure S2d+f-h)

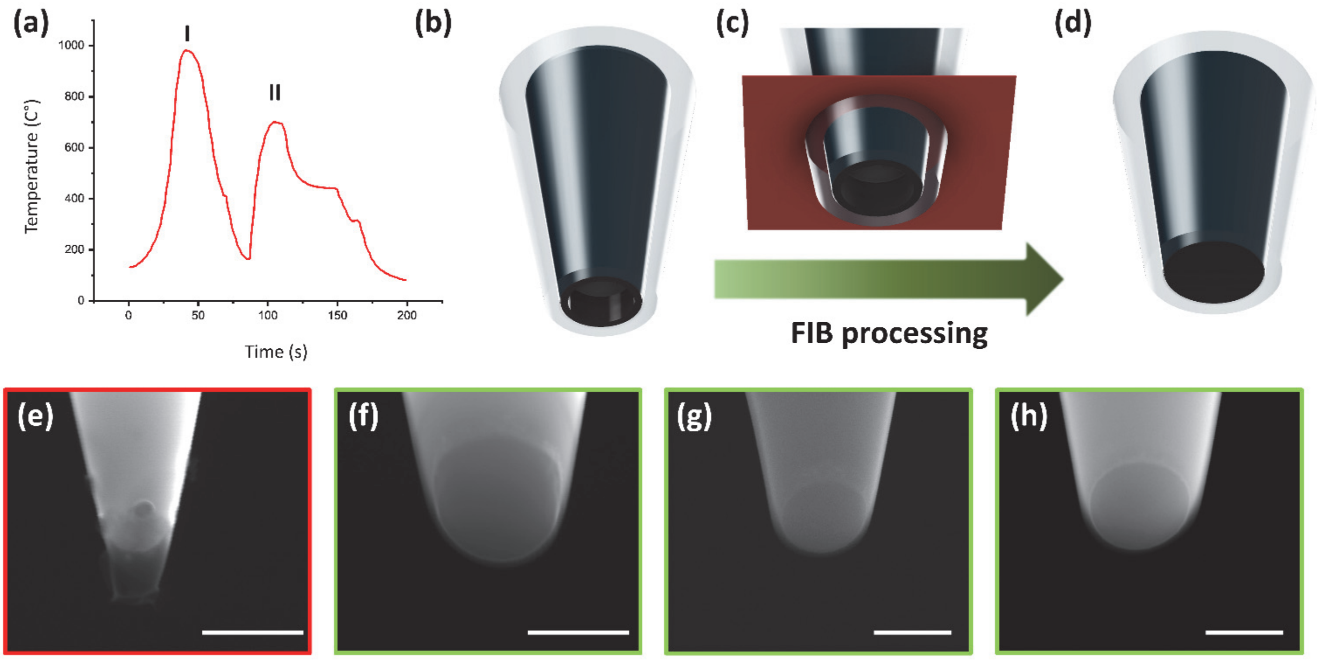

Figure S2: **(a)** Pyrolysis profile during the pyrolysis of the butane/propane gas mixture. Illustration of **(b)** the as-prepared recessed CNE. **(c)** The FIB plane is indicated in red at the position where the as-prepared CNE is trimmed. **(d)** Illustration of the obtained disk-shaped CNE. SEM micrographs of **(e)** an as-prepared CNE and **(f-h)** three different disk-shaped CNEs obtained after the FIB milling process. Scale bars equal 500 nm.

Section 3: Surface modification of the CNE

The surface modification was optimized to find the best compromise between as little as possible electron transfer blocking effect and as much as possible interaction with the Co_3O_4 NP. First, the electro-oxidative surface grafting mechanism as shown in Figure S3a was carried out using cyclic voltammetry to determine the oxidation potential of the N-Boc-ethylenediamine at the CNE surface. The corresponding CV is shown in Figure S3b, where a weak oxidation peak is observed at around 1.5 V vs. Ag/AgCl/3M KCl, which can be assigned to the oxidation of the amino group under formation of the corresponding radical cation. A decrease in the current in the second cycle can be observed, which is a first indicator that the electron transfer is hindered by the modification layer grafted in the first CV. The blocking effect is more clearly visible when the CNE is characterized in a 0.1 M KCl solution containing 5 mM $[Ru(NH_3)_6]Cl_3$ as the redox mediator. The cyclic voltammograms shown in S3c before (black) and after (red) show a decrease in the current of about 90% after modification. Also, the shape of the CV is changing after modification; the sinusoidal-shaped curve, which is achieved before the modification of the CNE, cannot be obtained anymore. Instead, a more linear increase of current can be observed, which indicates that an additional resistance is introduced at the surface of the CNE, which is limiting the electron transfer. Since the determination of the intrinsic activity of the NP is the goal of this study, blocking of the electrode to this extent must be avoided. Therefore, the modification procedure was subsequently carried out via a pulse deposition route where a single pulse of 20 ms at +1.5 V vs. Ag/AgCl 3M KCl is applied. In the corresponding CVs in a 0.1 M KCl solution containing 5 mM $[Ru(NH_3)_6]Cl_3$ only negligible current decrease is observed and the sinusoidal shape of the CV can be maintained (Figure S3d). Both observations indicate that a lower resistance for the charge transfer was introduced by the film created via the pulse method modification.

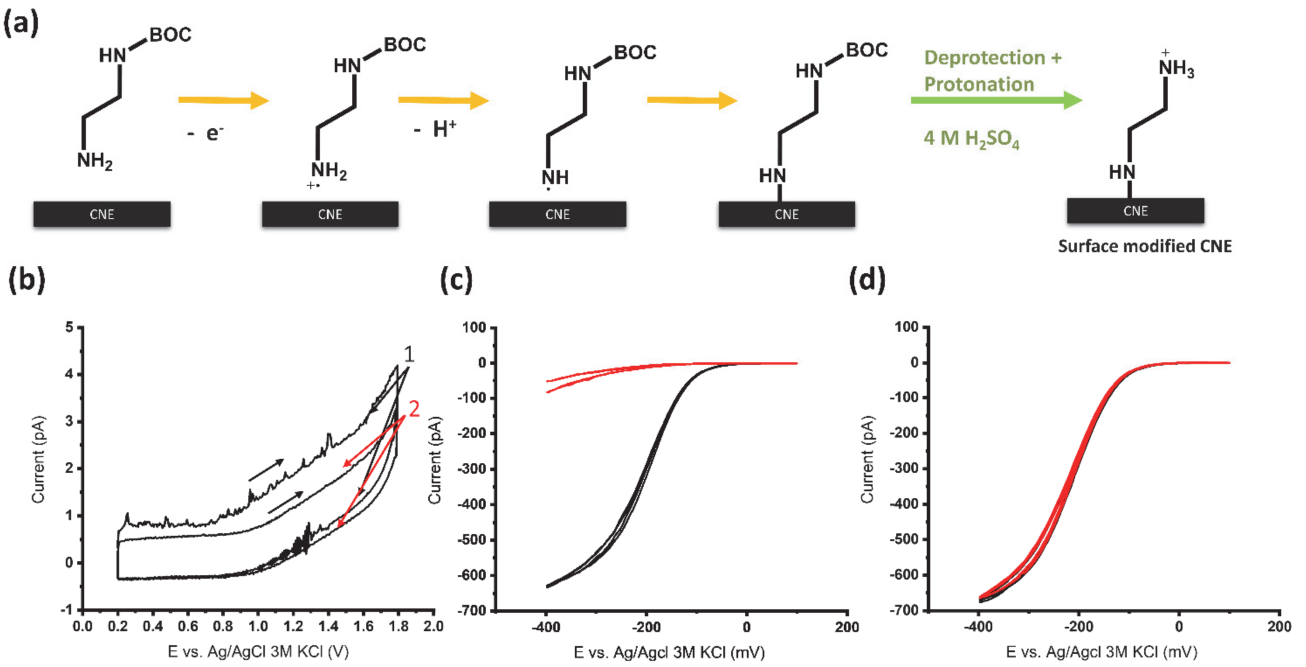

Figure S3: (a) An illustration of the electro-oxidative surface grafting mechanism. **(b)** Corresponding cyclic voltammogram of a CNE recorded in an ethanol solution containing 3.3 mM N-Boc-ethylenediamine solution and 0.1 M $LiClO_4$ as the supporting electrolyte at a scan rate of 50 $mV\cdot s^{-1}$.Two cyclic voltammograms were recorded and each voltammogram is labelled with the corresponding cycle number. **(c)** Characterization of the CNE modified in (b), the cyclic voltammograms were recorded in a 0.1 M KCl solution containing 5 mM $[Ru(NH_3)_6]Cl_3$ as redox mediator, before (black) and after surface grafting (red). **(d)** Characterization of a CNE modified with the pulse deposition method where a single pulse of 1.5 V vs. Ag/AgCl/3M KCl was applied for 20 ms to modify the CNE surface. The CVs were recorded under the same conditions as (c), before (black) and after modification (red).

The modified CNEs were dipped into absolute ethanol after the modification process to remove the excess of N-Boc-ethylenediamine. Subsequently, the CNE was immersed into a 4 M H_2SO_4 to remove the BOC protection group and simultaneously protonate the resulting NH_2 group. The CNEs were thereafter dipped shortly into absolute ethanol to remove the excess of H_2SO_4.

Section 4: Micromanipulator set-up

The micromanipulator set-up is depicted in Figure S4. The micromanipulator consists of the robotic arm which can be moved three-dimensionally in the x, y, and z-direction. The sample is mounted planar onto the stage of the SEM and the CNEs are positioned vertically to the tip, so that the disk-shaped surface of the CNE is collinear to the tip. Different probe tips differing in their tip radii can be attached to the robotic arm.

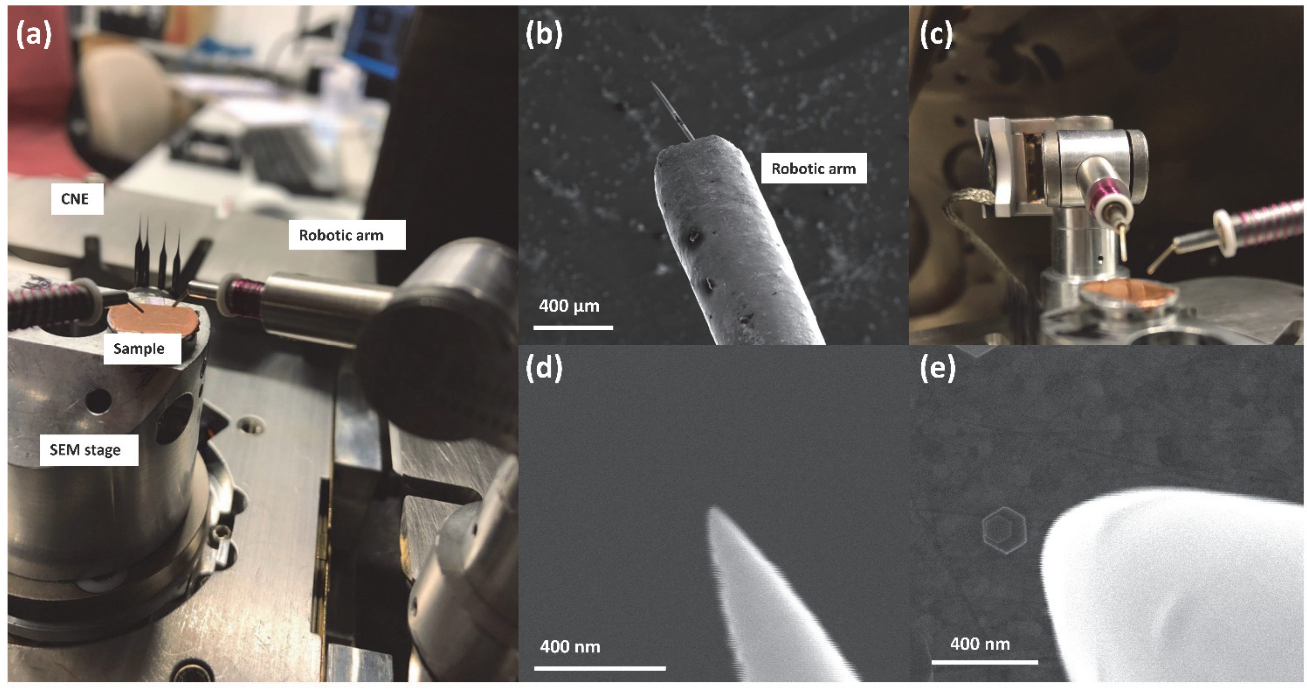

Figure S4: **(a)** and **(b)** Photographs of the micromanipulator set-up installed in the SEM chamber. **(c)** The probe tip which is mounted at the micromanipulator. **(d)** Probe tip with a tip radii of 20 nm. **(e)** Probe tip with a tip radii of 200 nm.

Section 5: Particle activation

The as-prepared nanoassembly initially did not display any electrochemical activity, however, after TEM investigation the particle was found to be active towards catalysing the OER (Figure S5b). The activation under the influence of the TEM beam can be possibly attributed to the removal of the capping agent oleylamine. The capping agent, which is used during the synthesis, acts as a stabilizer and yields a monodispersed NP solution, as desired for the present workflow. In order to establish a protocol which avoids the harsh conditions of the electron beam, removal of oleylamine from the NP surface by means of heat treatment was investigated. Co_3O_4 nanoparticles were placed on the surface of a CNE. The prepared nanoassembly was heated in an electric oven for 2 h at 200 °C in air. This temperature is reported to be high enough to remove the oleylamine completely from the particle surface.[4] In Figure S5 it is observed that the prepared nanoassembly exhibits poor activity towards the OER. However, similar to the observation after the TEM investigation, the nanoassembly shows a high electrocatalytic activity after the heating process.

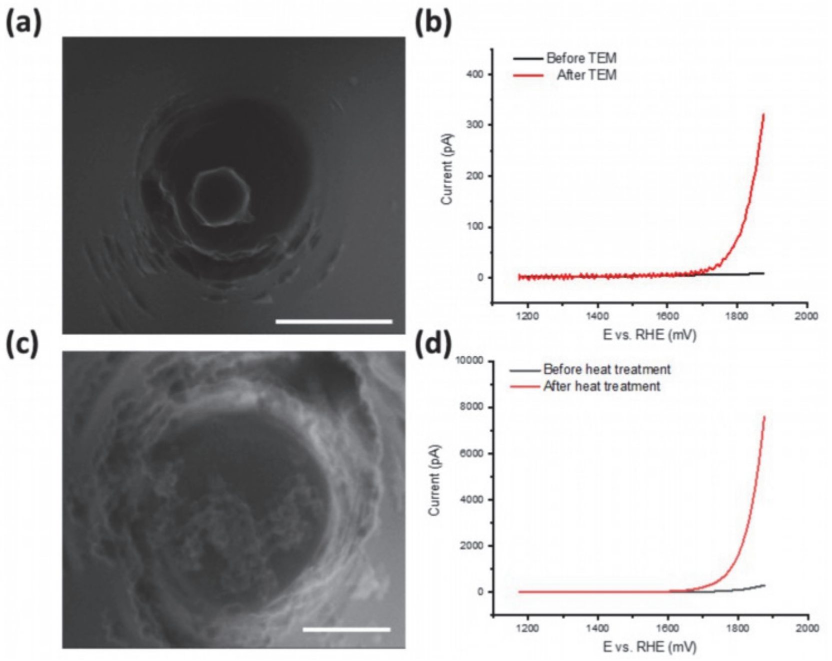

Figure. S5: (a) SEM image of the as-prepared nanoassembly. **(b)** Linear sweep voltammograms before (black) and after (red) particle investigation under the TEM beam, LSVs are recorded with a scan rate of 200 mV·s^{-1} in 0.1 M KOH. **(c)** SEM micrograph of a nanoassembly with a higher density of particles. **(d)** Corresponding LSVs before (black) and after (red) heat treatment. LSVs are recorded with a scan rate of 200 mV·s^{-1} in 0.1 M KOH. Scale bars equal 500 nm.

Section 6: Determination of the size-dependent electrochemical activity

6.1 Determination of the electrochemically active surface area and the volume

The surface area and the volume of the hexagon are calculated using the following equations, where a is the side length of the hexagon and h the thickness (height) of the hexagon (as indicated in Figure S6).

Equations:

The surface area of a hexagon: $6 \times a \times h + 2 \times \frac{3\times\sqrt{3}\times a^2}{2}$

Volume of a hexagon: $\frac{3\times\sqrt{3}\times a^2}{2} \times h$

Contact plane planar: $\frac{3\times\sqrt{3}\times a^2}{2}$

Contact area perpendicular: $a \times h$

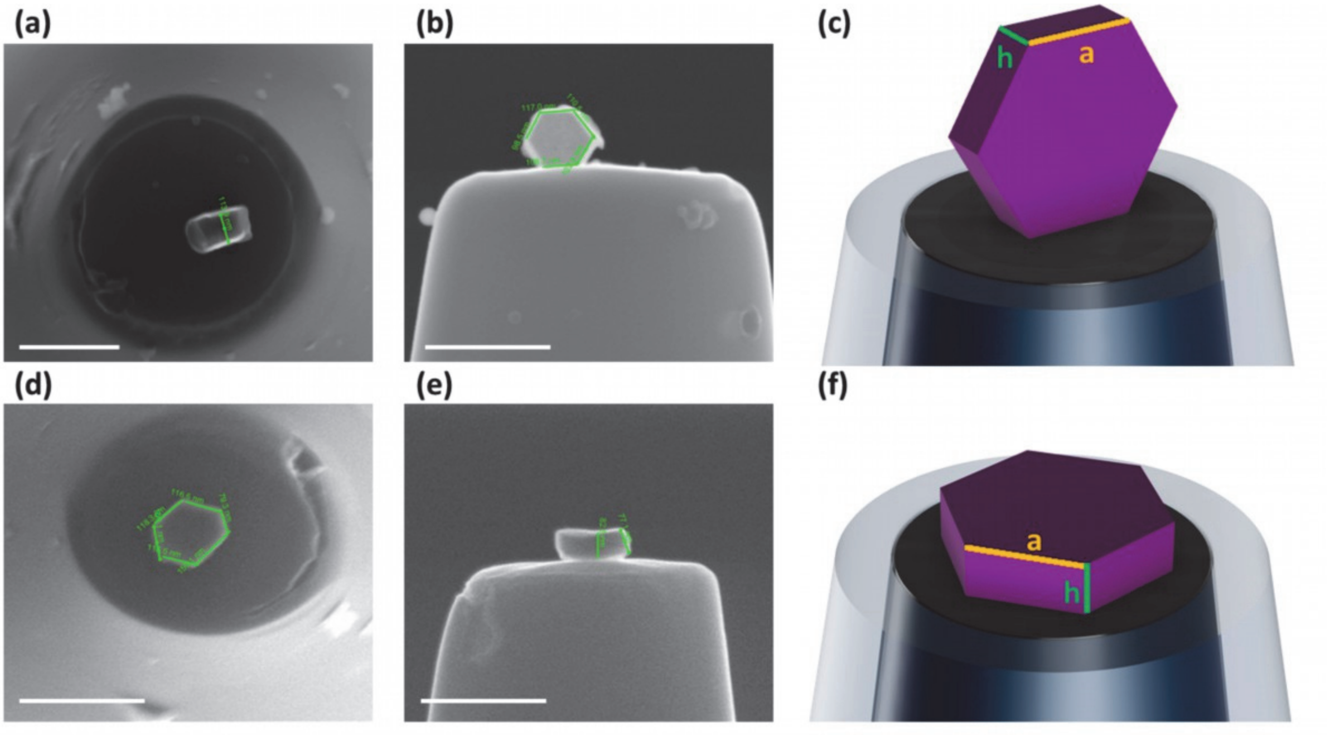

Figure S6| SEM image of an **(a)-(c)** perpendicular **(d)-(f)** planar placed hexagonal-shaped NP and the corresponding illustrations.

The electrochemical active surface area was calculated assuming that the contact side of the NP is not electrochemically accessible and thus can be subtracted for the current normalization. The following equations were obtained by subtracting the contact plane for the planar placed particles:

Electroactive surface area of a planarly placed Hexagon: $6 \times a \times h + \frac{3\times\sqrt{3}\times a^2}{2}$

The electrochemically active surface area was calculated by measuring the size of the NP on the nanoassembly by SEM from two different sides to get the information about the side length and the thickness of each individual hexagon. Due to the specific orientation in the TEM, only the thickness of the planarly arranged nanoassembly could be double-checked at the higher resolution of the TEM.

|**Table 1:** Calculated values of the surface area A and the Volume V for each CNE shown in the manuscript

Electrode No.	Side length a (nm)	Thickness h (nm)	Surface area A (cm ^{2})	Electroactive surface area A (cm ^{2})	Volume V (cm^3)
**CNE - 1**	160	50	1.81 x 10^{-9}	1.14 x 10^{-9}	3.33 x 10^{-15}
**CNE - 2**	107	56	9.54 x 10^{-10}	6.57 x 10^{-10}	1.67 x 10^{-15}
**CNE - 3**	113	70	1.14 x 10^{-9}	8.06 x 10^{-10}	2.32 x 10^{-15}

6.2 Determination of the turnover frequency (TOF)

The turnover frequency is calculated using the following equation:

$$TOF = \frac{j \times S}{4 \times n \times F}$$

Where j is the measured current density at a certain potential, S is the electrochemically active surface area of the nanoassembly, 4 is the number of transferred electrons, n is the number of moles of cobalt atoms present in the nanoassembly and F the Faraday constant (96485 C mol^{-1})

We used two different ways to obtain the number of moles of cobalt atoms which are considered to act as active sites for the OER. The first approach is more conservative assuming that all Co atoms in the particle are participating in the reaction. The second approach is based by considering only the Co atoms which are located at the surface of the NP.

6.2.1 TOF calculation: All cobalt atoms of the whole particle are participating in the reaction

The moles of cobalt atoms in the nanoassembly was calculated using a conservative approach, assuming that all cobalt atoms of the NP are participating in the reaction. Thus, a TOF number is calculated, which is the lowest and most conservative estimate for the particle.

$$n = moles\ of\ cobalt = \frac{density\ of\ Co_3O_4 \times Volume}{molar\ mass\ of\ Co_3O_4} \times mass\ percent\ of\ Co\ in\ Co_3O_4$$

The density of Co_3O_4 is 6.11 g cm^{-3} and the molar mass is 240.8 g mol $^{-1}$. The mass ratio between cobalt and oxygen is 73.42 % to 26.58 %.

Table 2: Calculated TOF values for the CNEs shown in the manuscript.

Electrode No.	Volume V (cm^3)	Current density j at 1.8 V (A/cm^2)	Current density j at 1.92 V (A/cm^2)	TOF at 1.8 V (s^{-1})	TOF at 1.92 V (s^{-1})
**CNE - 1**	3.33 x 10^{-15}	0.6	11.2	37.8	539.7
**CNE - 2**	1.67 x 10^{-15}	0.7	11.5	55.7	629.4
**CNE - 3**	2.32 x 10^{-15}	0.6	8.9	34	429.3

The obtained values were averaged and the resulting TOF value and the corresponding standard deviation is 33 ± 5 / 532 ± 100 s^{-1} at 1.8 V / 1.92 V vs. RHE.

6.2.1 TOF calculation: Only the Co atoms located at the nanoparticle surface are participating in the reaction

In a first step, the atom density in the two different exposed surface planes is calculated using the crystallographic software VESTA for counting the Co atoms in the different exposed surfaces planes of the nanoparticle.

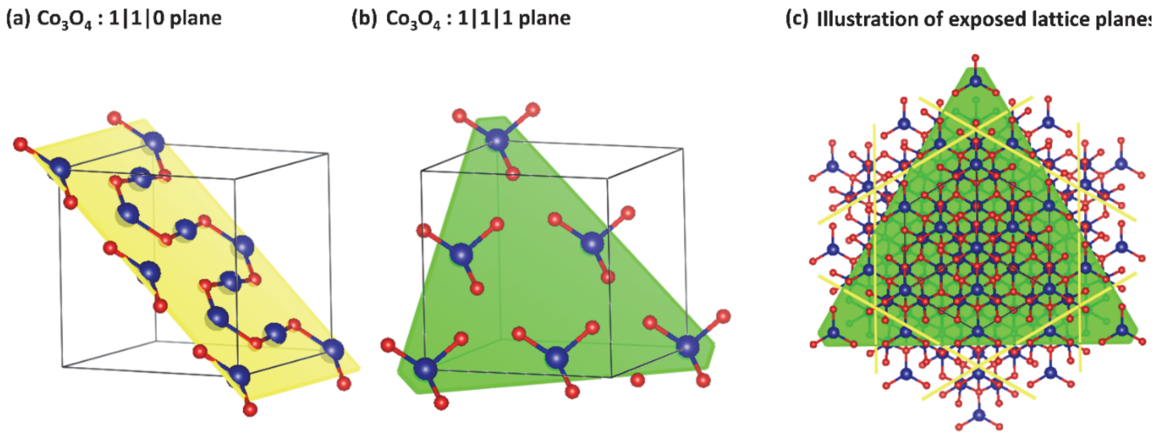

Figure S7: Illustration of surface atoms in the two different present planes **(a)** 1|1|0 **(b)** 1|1|1 of the particle, where blue represents the Co atoms and red the O atoms The number of atoms in these planes is in agreement with literature.[5] Illustration of all exposed sites of a hexagon shaped nanoparticle (1|1|0 indicated in yellow and 1|1|1 indicated in green) **(c)**.

Based on Figure S7, the number of exposed Co atoms can be calculated considering that 2D planes make up the surface of the particle. To form the top surface of the hexagonal particle, the 1|1|1 plane (Figure S7b) is needed six times. Therefore, the atoms on the vertices of the unit cell are shared by six adjacent planes. The atoms on edges are shared by two planes. The side walls of the hexagonal particle are formed by six 1|1|0 planes (Figure S7a+c). Due to its squared shape, the atoms on the vertices are shared by four planes, whereas the atoms on the edges of the unit cell are shared by two planes. The inner atoms are counted as full atoms.

Co atoms exposed at the 1|1|0 facet: $N_{1|1|0}4 \times \frac{1}{4} + 2 \times \frac{1}{2} + 6 \times 1 = 8$

Co atoms exposed at the 1|1|1 facet: $N_{1|1|1} = 3 \times \frac{1}{6} + 3 \times \frac{1}{2} = 2$

By using the known size of the unit cell (8.065 Å) one can calculate the atom density for each plane:

Atom density at the 1|1|0 facet: $Area_{1|1|0} = \sqrt{2} \times (8.065\ \text{Å})^2 = 9.199^{-19} m^2 \quad atom\ denisty = \frac{number\ of\ cobalt\ atoms}{Area\ of\ the\ facet} = 8.697 \times 10^{18} \frac{Co\ atoms}{m^2}$

Atom density at the 1|1|1 facet: $Area_{1|1|1} = \frac{\sqrt{3}}{2} \times (8.065\ \text{Å})^2 = 5.633^{-19} m^2 \quad atom\ denisty = \frac{number\ of\ cobalt\ atoms}{Area\ of\ the\ facet} = 3.5505 \times 10^{18} \frac{Co\ atoms}{m^2}$

The number of moles of cobalt atoms located at the surface can now be calculated by considering the different exposed surface areas, namely one 1|1|1 hexagonal-shaped exposed surface area at the top and six times the 1|1|0 exposed rectangle surface areas at the sides of the nanoparticle, where N_A is the Avogadro constant.

$$n = Moles\ of\ Co\ atoms = \frac{atom\ denisty_{1|1|1} \times Surface\ area_{1|1|1} + atom\ denisty_{1|1|0} \times 6 \times surface\ area_{1|1|0}}{N_A}$$

Table 3: Calculated TOF values for the CNEs shown in the manuscript by exposed surface Co atoms.

Electrode No.	Co atoms (mol)	Current density j at 1.8 V (A/cm^2)	Current density j at 1.92 V (A/cm^2)	TOF at 1.8 V (s^{-1})	TOF at 1.92 V (s^{-1})
**CNE - 1**		0.6	11.2	1640.3	30700
**CNE - 2**		0.7	11.5	1715.5	28233
**CNE - 3**		0.6	8.9	1553.3	21130

The obtained values were averaged and the resulting TOF value and the corresponding standard deviation is 1636.4 ±81 / 26687 ± 4968.6 s^{-1} at 1.8 V / 1.92 V vs. RHE.

Section 7: TEM characterization post electrochemical study

After the electrochemical testing, the nanoassembly is evaluated by means of TEM. STEM images and corresponding EDS elemental maps of the nanoassembly are shown in Figure S7. The elemental maps of the nanoassembly indicate that in the Co_3O_4 NP the oxygen content is significantly increased in direct proximity to the particle surface. This is well observable in the line scan profile (Figure S7d), where two peaks in the oxygen spectra are visible at around 32.6 nm and 96.6 nm away from the start position. The first one is corresponding to the increased amount of oxygen at the surface of the NP, which is indicating a possible formation of a cobalt oxide/oxyhydroxide layer. The second peak can be attributed to the an overlapping increase in the Si content arising from the exposed quartz-based nano capillary surface accompanied with the decrease in the cobalt content.

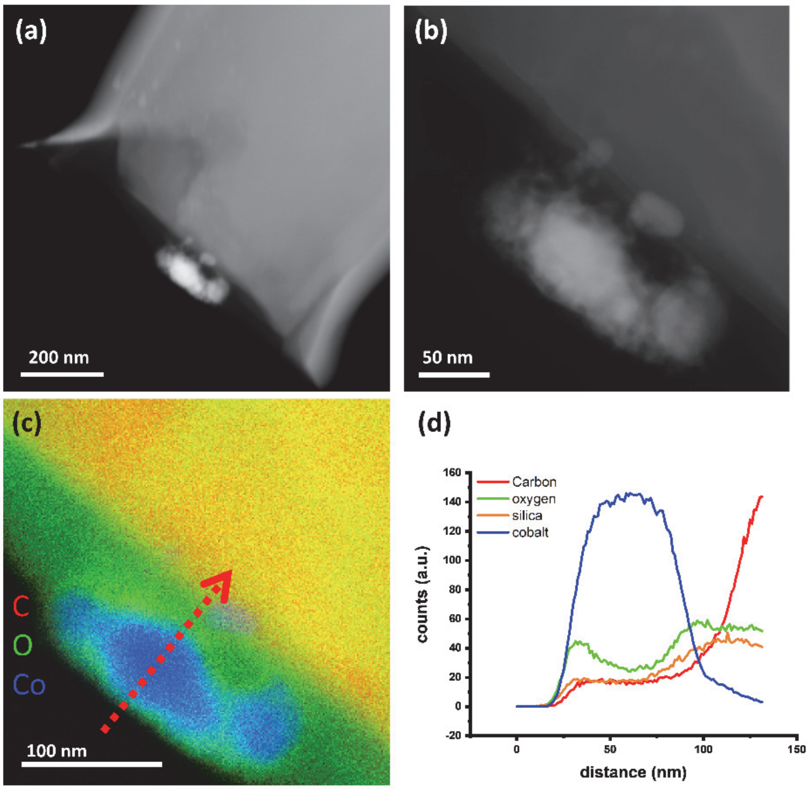

Figure S7: **(a)** and **(b)** STEM micrographs of the Co_3O_4 - CNE nanoassembly shown in Figure 3b in the manuscript. **(c)** Corresponding EDS elemental mapping of the particle, cobalt (blue), Oxygen (green), and carbon (red). **(d)** The line scan profile of measured in (c) scan direction and the location is indicated by the red arrow.

Section 8: Stability evaluation of the prepared nanoassembly

The stability measurement was carried out amperometrically at a fixed potential. First, a potential of +1.17 V vs. RHE was chosen, where no electrocatalytic activity was observed. Subsequently, a potential of +1.77 V vs. RHE was applied for 50 to 60 seconds to investigate the stability of the prepared nanoassembly during the course of the oxygen evolution reaction. During these measurements, the importance of modifying the CNE with N-Boc-ethylenediamine previous to the particle placement and activation was underlined. The chronoamperogram recorded using surface modified CNEs (prior to their activation) exhibits a stable catalytic current over 50s (Figure 8a). In contrast, the non-modified nanoassembly displayed an unstable current, indicative of poor electric conductivity between the particle and the CNE surface or a partial loss of connection.

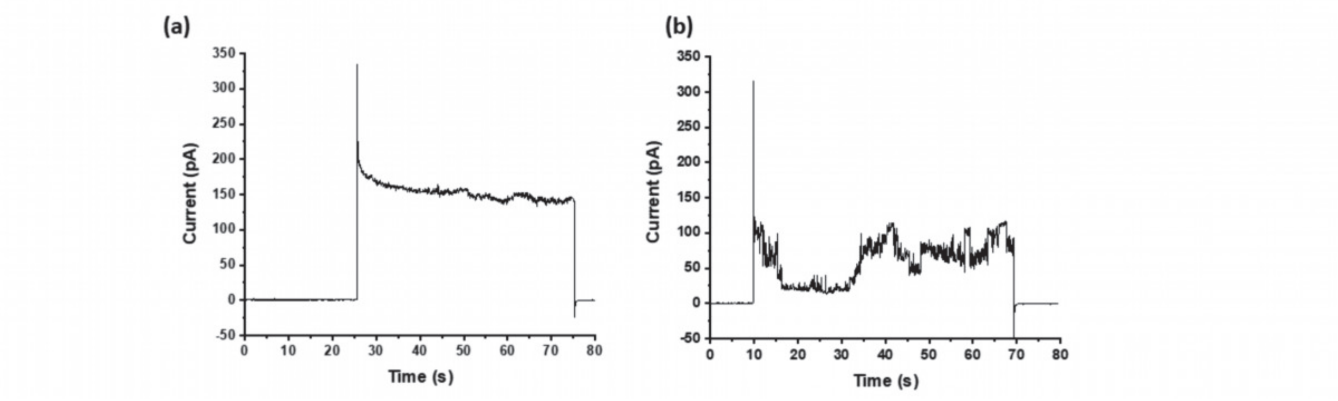

Figure S8: Chronoamperograms of **(a)** a nanoassembly which was modified previously to the NP placement and **(b)** which was not modified previous to the NP placement. Both chronoamperograms were started at a potential of +1.17 V vs. RHE and then the potential was increased to +1.77 V vs. RHE before it was again switched back to 1.17 V vs. RHE after 50 to 60 seconds. Both nanoassemblies were measured in 0.1 M KOH.

References

[1] S. Saddeler, U. Hagemann, S. Schulz, *Inorg. Chem.* **2020**, *59*, 10013.
[2] X.-Y. Yu, Q.-Q. Meng, T. Luo, Y. Jia, B. Sun, Q.-X. Li, J.-H. Liu, X.-J. Huang, *Scientific Reports* **2013**, *3*, 2886.
[3] P. Wilde, T. Quast, H. B. Aiyappa, Y.-T. Chen, A. Botz, T. Tarnev, M. Marquitan, S. Feldhege, A. Lindner, C. Andronescu, W. Schuhmann, *ChemElectroChem* **2018**, *88*, 414.
[4] D. Li, C. Wang, D. Tripkovic, S. Sun, N. M. Markovic, V. R. Stamenkovic, *ACS Catal.* **2012**, *2*, 1358.
[5] D. Liu, X. Wang, X. Wang, W. Tian, Y. Bando, D. Golberg, *Scientific Reports* **2013**, *3*, 2543.

Author Contributions

T. Quast: Data collection (lead), data interpretation (equal),
H. B. Aiyappa: Data interpretation (equal), writing of initial draft (lead)
S. Saddeler: Data collection (supporting), data interpretation (supporting)
P. Wilde: Data collection (supporting), data interpretation (supporting)
Y.-T. Chen: Data collection (supporting), data interpretation (supporting)
S. Schulz: Project administration (equal), funding acquisition (equal), data interpretation (equal)
W. Schuhmann: Research idea and concept (lead), project administration (equal), funding acquisition (equal), data interpretation (equal), writing of manuscript (supporting)
